# Trauma research forum

**DOI:** 10.1186/1757-7241-20-S1-I4

**Published:** 2012-03-22

**Authors:** Thomas Kristiansen, Kjetil G Ringdal

**Affiliations:** 1 Norwegian Air Ambulance Foundation, Department of Research, Drøbak, Norway; 2 Institute of Clinical Medicine, Faculty of Medicine, University of Oslo, Norway

## 

Parallel to the start of this year’s London Trauma Conference, a session dedicated to trauma research was introduced and hosted by Professor Karim Brohi. Titled “London Trauma Research Forum”, the program was devised as a *day showcasing trauma research across the London Trauma System*. In his opening address Prof. Brohi emphasised that the development of the London trauma system is a unique opportunity to strengthen trauma research. The resources spent on trauma related research compare poorly to other fields of medicine and are not in proportion to the resources devoted to the clinical management of trauma - by far. This is disappointing as trauma is the only current disease in the UK with an increasing mortality rate. The propensity for trauma to affect young adults, adds to the societal burden of this disease. The evidence base and the quality of research for the management of severe trauma is not impressive, and while the annual rate of academic contribution to this clinical field may be beyond the scope of a journal’s special issue, it probably wouldn’t overwhelm any librarian. However, to criticise the allocators of resources for this imbalance may be misplaced. On the contrary, the main issue may be a lack of academic initiatives from the trauma community itself; well formulated applications describing well founded topics, high quality projects and large scale trials, will secure funding for research related to trauma as it secures resources for research in any other field of medicine. There are many reasons why such applications are scarce from the trauma community. Trauma is an acute disease often occurring in a chaotic environment where issues such as informed consent, randomization and detailed documentation may be in direct ethical or time dependent contrast to effective clinical management.

As the new London trauma system (http://www.londontraumaoffice.nhs.uk/) is developing into the largest formalized network of trauma care in the world, this should be paired with expectations of evening out the imbalance between the societal burden of trauma and the efforts towards improving trauma care. The London trauma system will have mandatory data admission to the TARN database (https://www.tarn.ac.uk/). This will secure a unique combination population-based and detailed clinical data from an extensive population. This, combined with the ability to follow a patient’s course through all the phases of trauma care within the system, enables clinical trials and observational studies with a magnitude and with a closed-loop quality that is truly unique.

It is evident from the speakers in the research symposium that it is not just patient data which is united with the development of a trauma system. Diversity is a key word describing the research forums topics and researchers. The first session, titled “Systems Research & Clinical Trials” illustrated how current projects that have already reached clinical trial stage, may benefit from the higher patient volume following the centralization of trauma management. An example was given by Dr Howard Levy, who is currently conducting a stage 2b trial on a novel oxygen delivery compound. The study requires 300 carefully selected trauma patients.

A trauma system should be under continuous audit and quality improvement. Tracy Parr from the London Trauma Office presented preliminary results assessing the precision of the newly implemented triage tool for pre-hospital personnel. In addition to testing the performance of the triage tool, the study has revealed important challenges and insights into linking data captured at the different phases of management – the necessity of a unique patient identification number is a lesson most certainly applicable to other regions with trauma system aspirations.

How do we ensure that the research that is conducted leads to improved care? How much evidence do we need to implement, say, tranexamic acid in our treatment protocols? The implementation of research is an important topic. The London Haematology & Trauma Group is a multi professional organisation with clinicians and researchers. The group aims to conduct research and standardise management in transfusion medicine across the trauma system. The newly formed group has already reviewed the trauma system’s transfusion guidelines and emphasised issues of communication and logistics in massive transfusion. The session on bleeding and blood transfusion introduced studies from both within and beyond the hospitals of London. Trauma research fellow Sirat Kahn presented his project on the systematic evaluation of the use of current massive haemorrhage trauma protocols. Mr Kahn also form part of the research group lead by Prof. Brohi which is responsible for the multicentre and multinational ACIT II study, applying thromboelastometry in trauma care. Gathering data from numerous leading trauma centres worldwide, the ACIT II study is an example of the feasibility and importance of research networks that extend beyond even the largest of clinical networks.

The diversity of the trauma related research linked together by the London trauma system is mentioned. To learn that the salmon canapés served for lunch may protect our neurones in a traffic accident was perhaps as surprising as the fact that there exists a parallel virtual universe where thousands of people interact daily and which is fully capable of hosting mass casualty training sessions. Prof. Adina Michael-Titus was one of several experimental and translational researchers presenting to the forum participants. Her research on the effect of DHA (Docosahexaenoic acid – an omega-3 fatty acid abundant in fish oils) on injured rodent neurones was convincing to the point that a proposed study on HEMS delivery of omega-3 capsules to traumatic brain injury (TBI) patients, seemed like a natural future (although perhaps not *next*) step. Prof. Christop Thiemermann’s studies on the organ protective effect of EPO (Erythropoietin) in injured rodents, was another excellent example of experimental research with potentially future implications for trauma management.

Dr. Tolias’ talk on innovative research in neurotrauma emphasised the need for novel research design. Microdialysis and PET scans were key words in this presentation and the lack of TBI studies in the pre-hospital setting was also pointed out. Combining translational research with innovative designs for clinical studies, TBI may very well represent the next frontier in trauma research.

The use of computer simulation in training may be cost effective and in mass casualty incidents, it may allow for preparedness in cases where improvisation may be the only alternative. Daniel Cohen from Imperial College, London has done a feasibility study using the online virtual resource Second Life (secondlife.com) in training for e.g. incidents involving biohazards. By programming in this virtual environment, participants can be tested in almost an unlimited number of scenarios regardless of their geographical location.

As much as a trauma system is not complete without a rehabilitation service, a forum on trauma research is not complete without a discussion on outcome measures. Working in the field of rehabilitation, occupational therapist Karen Hoffman, presented several well established rehabilitation indexes and how these may perform both as predictors of severity and measures of outcome.

Trauma is a diverse disease; the London trauma research forum suggested that the academic efforts required to improve trauma care need to be equally diverse. In the forum’s final session Prof. Brohi presented the Masters in Trauma Sciences program, which is launched this year. This collaboration between the Royal College of Surgeons of England and the Queen Mary College University of London adds to the impression that there is an academic upgrading coupled to the development of the London Trauma System. Strengthening trauma research is a challenge and a responsibility for the international trauma community in the years to come. The lesson from London may be that the development of a trauma system can help shift the imbalance between the burden of trauma and the efforts towards improving trauma.

